# County-Level Analysis of the Health Impact of Black Physicians in Georgia

**DOI:** 10.1007/s40615-025-02423-8

**Published:** 2025-04-16

**Authors:** Chaohua Li, Peter Baltrus, Robina Josiah Willock, Mitchell Blount, Anne Gaglioti, Bryant Bailey, Lee S. Caplan, Megan D. Douglas, Dominic Mack

**Affiliations:** 1 National Center for Primary Care, Morehouse School of Medicine, 720 Westview Dr SW, Atlanta, GA 30310 USA; 2 Department of Community Health and Preventive Medicine, Morehouse School of Medicine, 720 Westview Dr SW, Atlanta, GA 30310 USA; 3 Department of Family Medicine, Morehouse School of Medicine, 720 Westview Dr SW, Atlanta, GA 30310 USA

**Keywords:** Physician workforce, Representativeness, Racial disparity, Years of potential life lost, Hospital discharges, Emergency room visits

## Abstract

Despite the longstanding underrepresentation of Black physicians in the U.S., greater representation of Black physicians in the physician workforce can positively impact health outcomes. In Georgia, racial and ethnic health inequities are prevalent, and physician workforce shortages are acute. This study aims to assess the impact of Black physician representation on three health outcomes: Years of Potential Life Lost (YPLL) before age 75 and hospital discharges and emergency room visits related to chronic Ambulatory Care Sensitive Conditions (ACSC) or coronavirus disease 2019 (COVID-19). Data sources included a survey administered by the Georgia Composite Medical Board and the Online Analytical Statistical Information System (OASIS) to analyze county-level outcomes for non-Hispanic Black (NHB) and non-Hispanic White (NHW) populations from 2016 to 2019 and 2020 to 2022. We used linear regression models to assess the association between Black physician representativeness in the county physician workforce and NHB-NHW disparities in the outcome measures. We found that counties with higher Black physician representativeness experienced better health outcomes for both NHB and NHW populations, with reduced racial disparities in hospital discharges and YPLL, particularly during the COVID-19 pandemic. The study underscores the importance of increasing Black physician representation in the workforce to advance health equity in Georgia.

## Background

The recent National Academies of Science, Engineering, and Medicine report, “Ending Unequal Treatment,” found that the United States (U.S.) has made little progress in advancing racial healthcare equity in the last 20 years [1]. Representation in medicine, particularly of physicians from minoritized and underserved communities, plays a critical role in addressing long-standing and pervasive racial and ethnic health inequities [2, 3].

One key concept in understanding the impact of representation in medicine is physician–patient racial concordance, which refers to the racial or ethnic matching between a patient and his or her physician. Racial concordance has been linked to better communication and higher patient satisfaction which may indirectly contribute to better health outcomes [4–7]. Patients are more likely to trust and adhere to medical advice when treated by physicians with similar racial or ethnic backgrounds [8, 9].

Despite the benefit of racial and ethnic physician–patient concordance in the healthcare setting, Black populations remain uniquely disadvantaged in their access to racially and ethnically concordant healthcare. An estimated 13% of the U.S. population identifies as Black or African American, yet less than 6% of physicians in the U.S. identify as Black or African American [10]. This lack of representation perpetuates generalized mistrust of the healthcare system, poor physician–patient communication, and a lack of cultural understanding, which exacerbate persistent racial and ethnic health inequities [11, 12].

In addition to individual-level benefits associated with physician–patient concordance, physician representativeness is associated with positive workforce and population health outcomes. Primary care physicians who identify as Black, Native American, or Hispanic are more likely than their White counterparts to practice in areas federally designated as medically underserved or experiencing health professional shortages [13, 14]. Black physician representation in the primary care workforce was associated with increased life expectancy and reduced mortality rates among Black individuals in the U.S. [15].

Additional research is needed to examine the mechanisms through which Black physician representativeness improves population health and to assess state- and county-level variation in this association. This study aimed to assess Black physician representativeness in a single state, Georgia, and its association with several indicators of population health across two time periods, prior to and during the COVID- 19 pandemic. This study included years of potential life lost (YPLL), a measure of premature mortality that reflects the impact of early deaths on a population. In addition, this study included emergency room (ER) visits and hospitalizations, which are commonly used as intermediate indicators of population health and adequate access to primary care, and are critical indicators of outpatient management of acute and chronic health conditions. Finally, this study analyzed data from before and during the coronavirus disease 2019 (COVID- 19) pandemic, which disproportionately affected racial and ethnic and other underserved communities, to determine whether Black physician representativeness in Georgia was associated with population health and chronic condition outcomes during the pandemic.

Georgia serves as a strong case study for several reasons. First, Georgia has a long-standing history of intractable health inequities among its underserved and minoritized populations, with Black people comprising 33% of the state’s population [16–18]. Second, Georgia faces physician workforce shortages, meeting only 38% of its physician workforce need, and with approximately 3.3 million Georgians living in a primary care shortage area [19]. Finally, Georgia is home to one of only four Historically Black College and University (HBCU) medical schools in the country, Morehouse School of Medicine, whose mission is explicitly focused on the development of a representative workforce and advancing health equity. By examining the relationship between the representativeness of Black physicians and population health outcomes in Georgia, this research seeks to better understand the impact of Black physician representativeness on population-level health outcomes generally and during a global pandemic that disproportionately affected Black communities.

## Methods

The physician-level data used in this study was collected along with biennial 2019–2020 physician renewal data by the Georgia Board of Health Care Workforce [20]. These surveys were completed by physicians when they renewed their licenses to practice medicine with the Georgia Composite Medical Board (GCMB). All active Georgia physicians (100% response rate) completed the survey for the 2019–2020 cohort. Physicians self-reported their specialty, gender, race, and practice location. Physicians were excluded from this study if data on race was missing.

County-level health data were obtained from the Online Analytical Statistical Information System (OASIS) from the Georgia Department of Public Health [21]. OASIS is a web-based tool that allows researchers to access publicly available health data and statistics for the state of Georgia. The main predictor of interest was the Black physician representative ratio (BPRR) in a county. BPRR was calculated using the following equation:

$$BPRR={\textstyle\frac{\left(\frac{Number\;of\;Black\;physicians\;in\;a\;country}{Number\;of\;all\;physicians\;in\;a\;country}\right)}{\left(\frac{Black\;population\;in\;a\;country}{Total\;population\;in\;a\;country}\right)}}$$

The algorithm of BPRR was developed based on a study by Snyder et al. [15]. BPRR is 1.0 when the proportion of the physician workforce who self-identified as Black equals the proportion of community members in that county who identified as Black. A BPRR value greater than or less than 1.0 indicates overrepresentation or underrepresentation respectively, of Black individuals in the physician workforce relative to the community. As the BPRR value tends to be unstable when the number of all physicians in a county is small, we excluded counties with less than five physicians from the analysis.

There were three county-level health outcomes of interest:

1. Yearly YPLL before 75 years old per 1000 deaths (YPLL/1000 deaths).
2. Yearly number of hospital discharges per 100,000 people (discharges/100 k) with a primary diagnosis code of either a chronic Ambulatory Care Sensitive Condition (ACSC) or COVID- 19.
3. Yearly number of emergency room (ER) visits per 100,000 people (ER/100 k) with a primary diagnosis code of a chronic ACSC or COVID- 19.

YPLL is a statistic that serves as a measure of premature mortality by estimating how many years a person would have lived if they had not died early (before 75 years old). Due to the statistical reliability and confidentiality concerns, this outcome was set to be missing by the OASIS if the total number of deaths in the county in the year was less than five. Chronic ACSCs include conditions that can typically be addressed and managed through regular primary care treatment. Chronic ACSC conditions included angina, asthma, chronic obstructive pulmonary disease, congestive heart failure, diabetes with ketoacidosis or coma, diabetes with unspecified complications, diabetes without complications, grand mal and other epileptic conditions, hypertension, and pulmonary and non-pulmonary tuberculosis.

The three outcomes were calculated for the non-Hispanic Black (NHB) population, the non-Hispanic White (NHW) population, and the total population. Other race/ethnicity groups were not considered in this study due to the scarcity of physician or outcome data specific to these groups and including these groups would lead to a large amount of unstable or missing estimates on the county level. The outcomes based on chronic ACSC were calculated for two time periods, i.e., 2016–2019 and 2020–2022, while the outcomes based on COVID- 19 were calculated for 2020–2022 only. Differences in the three outcomes between NHB and NHW were also calculated to examine racial disparities in the outcomes.

County-level demographic data from the 2020 estimate of the 5-year American Community Survey included proportion population that was NHB, median age, proportion of people living under federal poverty level, and proportion of people without health insurance. A county was determined to be rural if the population was less than 50,000 according to the United States 2020 census. The number of hospitals in a county was obtained from Area Health Resources Files, U.S. Department of Health & Human Services [22].

Physician-level characteristics were calculated and reported by race, gender, primary care or non-primary care specialty, metropolitan status of the county, and age. Primary care specialties include general or family practice, general internal medicine, pediatrics, and obstetrics and gynecology. County-level characteristics and outcome measures were reported for two groups, BPRR < 1 and BPRR ≥ 1. Chi-square tests were conducted to compare the distribution of the rurality of the county as well as whether the county had at least one hospital, by BPRR group. Two-sample *t*-tests were performed to compare the rest of the county-level characteristics and outcomes by BPRR groups. We also created bar charts comparing the three outcomes across racial groups and counties with BPRR < 1 or BPRR ≥ 1 for each of the two time periods (2016–2019 and 2020–2022). Using BPRR < 1 as the reference group for the main predictor, we conducted linear regression models for the Black-to-White differences (Black minus White) in the three outcomes from 2020 to 2022. In the fully adjusted models, we adjusted for covariates including the proportion of the population that was Black, median age, the proportion of people living under the federal poverty line, and the proportion of people without health insurance to account for potential confounding from socio-demographic factors; we adjusted for rural status and whether the county had at least one hospital to account for potential confounding from physician and healthcare resource density. These covariates were selected a priori and included in the model based on theoretical relevance and prior empirical findings. No significant interactions were detected between the main predictor and each of the covariates, so interaction terms were not included in the final models. All *p*-values were two-sided, and a *p*-value < 0.05 was considered statistically significant. SAS version 9.4 (SAS Institute, Inc., Cary, NC, USA) was used to perform all analyses. Using ArcGIS software version 10.6, we created choropleth maps of the three outcomes for the all-races population and the non-Hispanic Black population in counties of Georgia from 2020 to 2022.

## Results

**Fig. 1:**
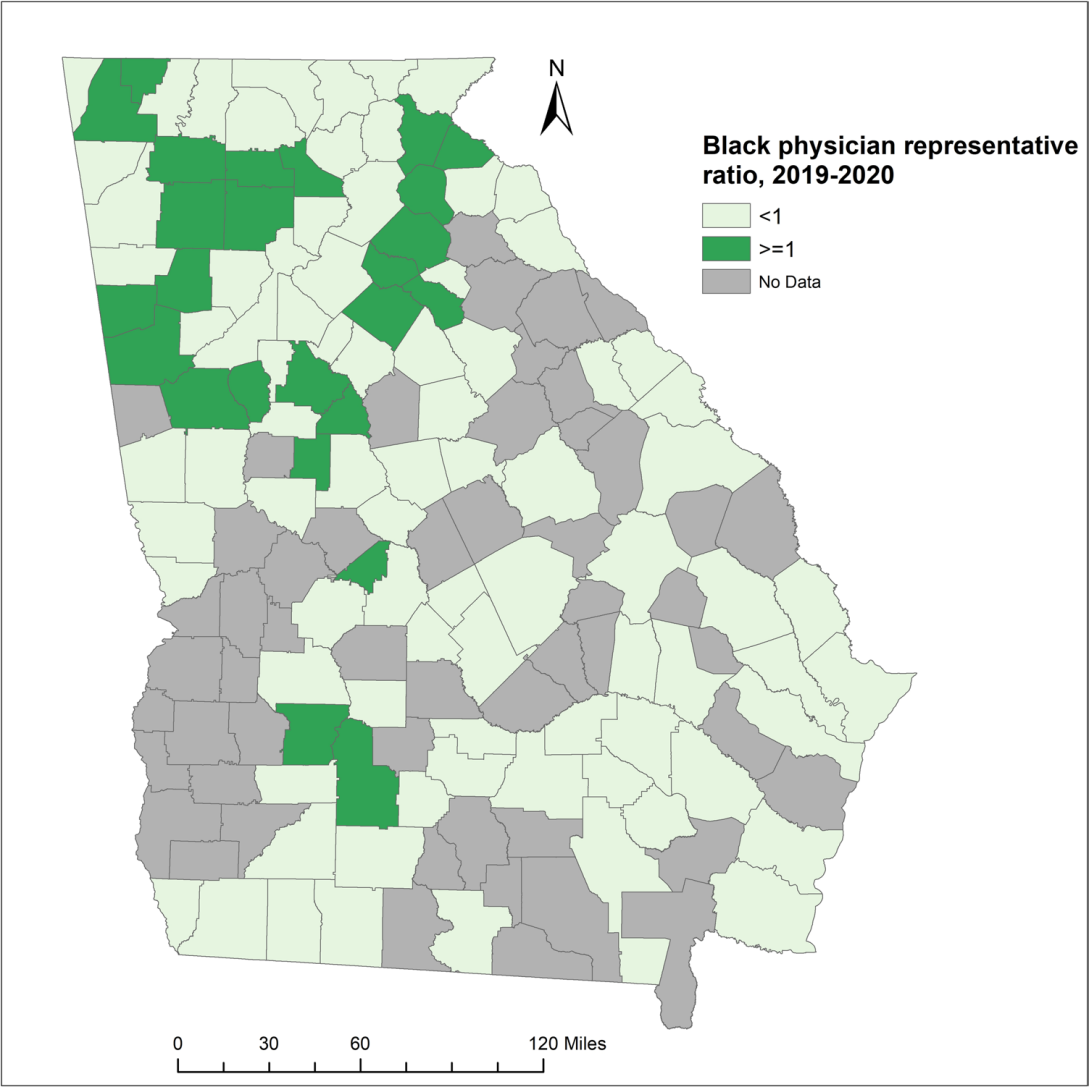
Black physician representative ratio for the counties in Georgia, 2019–2020

We included 15,036 physicians who completed the survey and provided responses for all key variables (Table 1). Among these physicians, 19.1% identified as Black, 32.2% identified as women, 37.6% specialized in primary care, and 10.8% practiced in rural counties. Compared to NHW physicians, Black physicians were more likely to be women (52.1% vs. 26.5%), less likely to specialize in primary care (35.9 vs. 38.2), less likely to practice in a rural county (6.6% vs, 12.2%) and were younger (51.7 years vs. 55.8 years). Geographically, the counties with BPRR ≥ 1 tended to be clustered together, and most of them were distributed in the north of Georgia, where the Metro Atlanta area is located (Fig. 1).

**Table 1:** Characteristics of physicians in Georgia, 2019–2020

		All races		White		Black		Asian		Other	
		*n*	%	*n*	%	*n*	%	*n*	%	*n*	%
Total		15,036	100	11,114	100	2870	100	898	100	154	100
Gender	Female	4837	32.2	2945	26.5	1495	52.1	337	37.5	60	39.0
	Male	10,199	67.8	8169	73.5	1375	47.9	561	62.5	94	61.0
Primary care physician	Yes	5653	37.6	4249	38.2	1031	35.9	328	36.5	45	29.2
	No	9383	62.4	6865	61.8	1839	64.1	570	63.5	109	70.8
Race	White	11,114	73.9	11,114	100						
	Black	2870	19.1			2870	100				
	Asian	898	6.0					898	100		
	Other	154	1.0							154	100
Practice in a rural county	Yes	1622	10.8	1356	12.2	190	6.6	63	7.0	13	8.4
	No	13,414	89.2	9758	87.8	2680	93.4	835	93.0	141	91.6
		mean	std	mean	std	mean	std	mean	std	mean	std
Age, years		54.5	12.2	55.8	12.2	51.7	10.7	48.7	12.6	47.4	11.9

*n* number, *%* column percentage, *std* standard deviation

**Fig. 2:**
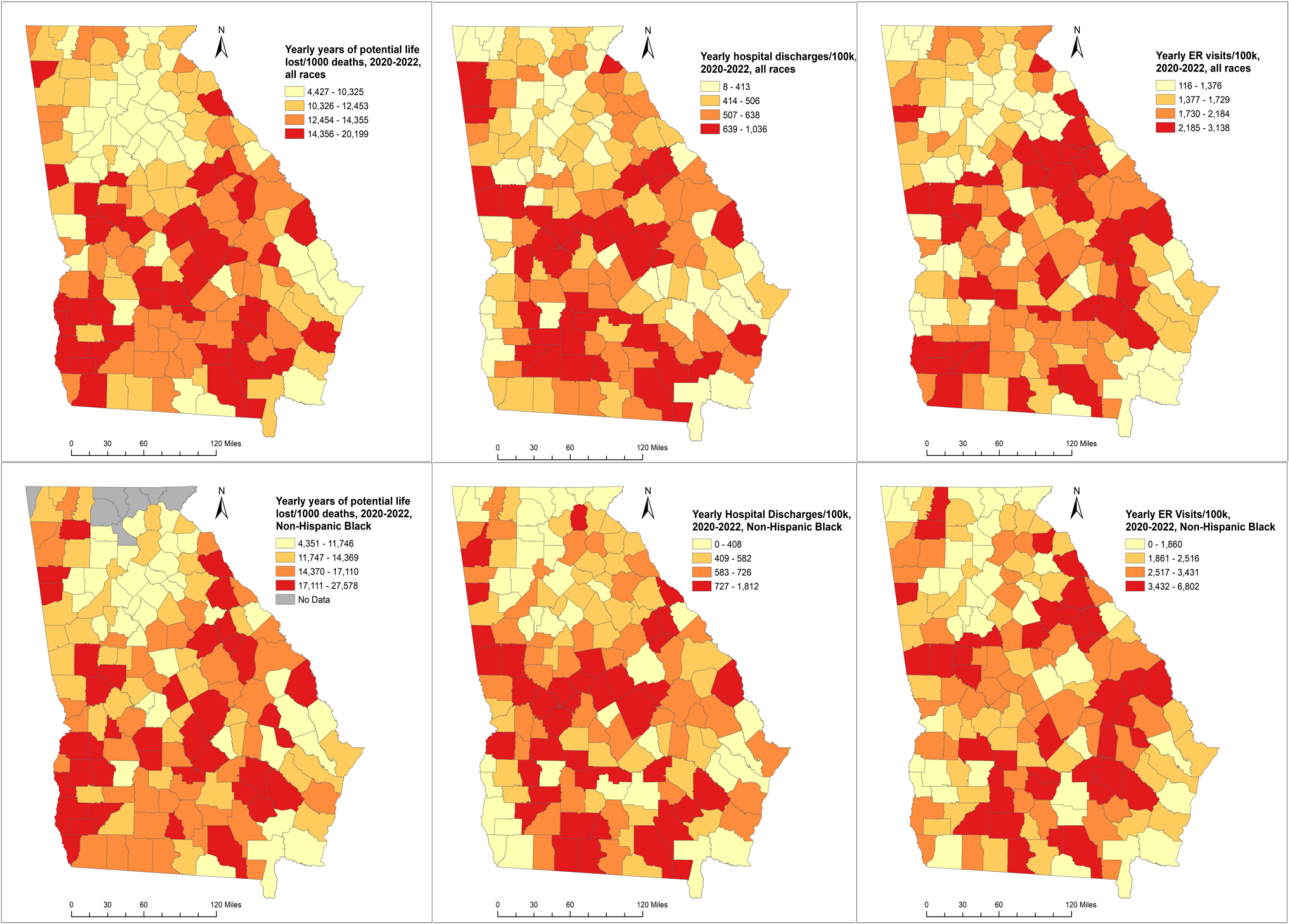
County rates of years of potential life lost, hospital discharges, and emergency room visits for all races and non-Hispanic Blacks in Georgia, 2020–2022

Figure 2 illustrates the geographical distribution of the three outcomes of interest for the total population and NHB population within counties in Georgia from 2020 to 2022. For all three outcomes, a similar pattern was observed for both the total population and the NHB population in which multiple clusters of counties with the highest rates were distributed in the south of the state, and the counties in the north generally had lower rates, especially those within the Metro Atlanta area.

We included 106 out of 159 Georgia counties in the analysis; 53 counties were excluded due to a total number of physicians in the county less than five. BPRR was < 1 in 81 counties (Black physicians underrepresented), and BPRR was ≥ 1 in 25 counties (Black physician overrepresented) (Table 2). Counties with BPRR ≥ 1 had lower proportion of Black population (15.8% vs. 29.1%), lower proportion of people living under the poverty line (12.3% vs. 18.2%), higher median income for Black population (30,872 vs. 26,289), lower proportion of rural counties (48.0% vs. 65.4%), and lower proportion of having at least 1 hospital (76.0% vs. 87.7%). Counties with BPRR ≥ 1 had consistently better outcomes, namely lower yearly hospital discharge rates, lower yearly ER visit rates, and lower yearly YPLL rates for both time periods across the total population, NHB population, and NHW population. In either BPRR group of counties, the NHB population had a higher ER visit rate and YPLL rate than the NHW population. Among counties with BPRR ≥ 1, the NHB-NHW disparity in the yearly hospital discharge rate due to chronic ACSC conditions was reduced from 74/100 k to 6/100 k, the disparity in the yearly hospital discharge rate due to COVID- 19 was reduced from 6/100 k to − 53/100 k, the disparity in the ER visit rate due to chronic ACSC conditions dropped from 1331/100 k to 974/100 k, the disparity in the ER visit rate due to COVID- 19 dropped from 806/100 k to 632/100 k, and the disparity in YPLL rate decreased from 1872/1000 to 404/1000 during 2020–2022.

**Table 2:** Characteristics of counties in Georgia by black physician representative ratio, 2020

	BPRR < 1 (*n* = 81)		BPRR ≥ 1 (*n* = 25)	
	Mean	std	Mean	std
%Black pop	29.1	17.6	15.8*	13.5
Median age	39.1	5.1	39.6	2.6
%Under federal poverty line	18.2	5.8	12.3*	4.4
%Uninsured	14.0	3.3	12.3*	2.6
Black median age	34.8	5.4	37.0*	5.0
Black %female	50.7	8.4	49.0	8.9
Black median income	26,289	5749	30,872	8380
Yearly hospital discharges for ACSC, chronic conditions (per 100,000 people)				
All Race (2016–2019)	694	195	560*	207
All Race (2020–2022)	518	160	440*	159
NHB (2016–2019)	712	305	491*	231
NHB (2020–2022)	578	282	452*	210
NHW (2016–2019)	647	253	507*	212
NHW (2020–2022)	505	178	446	157
Yearly ER visits for ACSC, chronic conditions (per 100,000 people)				
All Race (2016–2019)	2427	794	1918*	746
All Race (2020–2022)	1854	619	1411*	599
NHB (2016–2019)	3473	1604	2844	1268
NHB (2020–2022)	2699	1214	2223	920
NHW (2016–2019)	1719	728	1555	673
NHW (2020–2022)	1369	540	1249	535
Yearly years of potential life lost before age 75 (per 1000 deaths)				
All Race (2016–2019)	9438	1957	8472*	1888
All Race (2020–2022)	11,764	2584	10,147*	2348
NHB (2016–2019)	11,265	2641	9591*	2760
NHB (2020–2022)	14,013	3272	11,399*	3473
NHW (2016–2019)	9778	2245	8987	1946
NHW (2020–2022)	12,128	3004	10,925	2598
Yearly hospital discharges for COVID- 19 (per 100,000 people)				
All Race (2020–2022)	492	171	422	180
NHB (2020–2022)	484	237	378*	169
NHW (2020–2022)	478	176	431	187
Yearly ER visits for COVID- 19 (per 100,000 people)				
All Race (2020–2022)	1461	559	1272	559
NHB (2020–2022)	1933	1002	1759	948
NHW (2020–2022)	1127	507	1127	505
Racial inequities				
NHB-NHW 2020–2022 Discharges/100,000	74	230	6	141
NHB-NHW 2020–2022 ER/100,000	1331	947	974*	470
NHB-NHW 2020–2022 YPLL/1,000 deaths	1872	2922	404*	3082
NHB-NHW 2020–2022 COVID hospital discharges/100,000	6	191	− 53*	91
NHB-NHW 2020–2022 COVID ER/100,000	806	760	632	537
	*n*	%	*n*	%
Non-rural	28	34.6	13	52.0
Rural	53	65.4	12	48.0
At least 1 hospital	71	87.7	19	76.0
No hospital	10	12.3	6	24.0

*BPRR* Black physician representative ratio, *NHB* non-hispanic black, *NHW* non-hispanic white, *n* number, *%* column percentage, *std* standard deviation, *ER* emergency room visits due to chronic ambulatory care sensitive conditions; Discharges, hospital discharges due to chronic ambulatory care sensitive conditions

*Two-sample *t*-tests were conducted to compare continuous measures, and chi-square tests were conducted to compare categorical measures by BPRR groups, significant results (*p*-value < 0.05) were indicated by asterisks

**Fig. 3:**
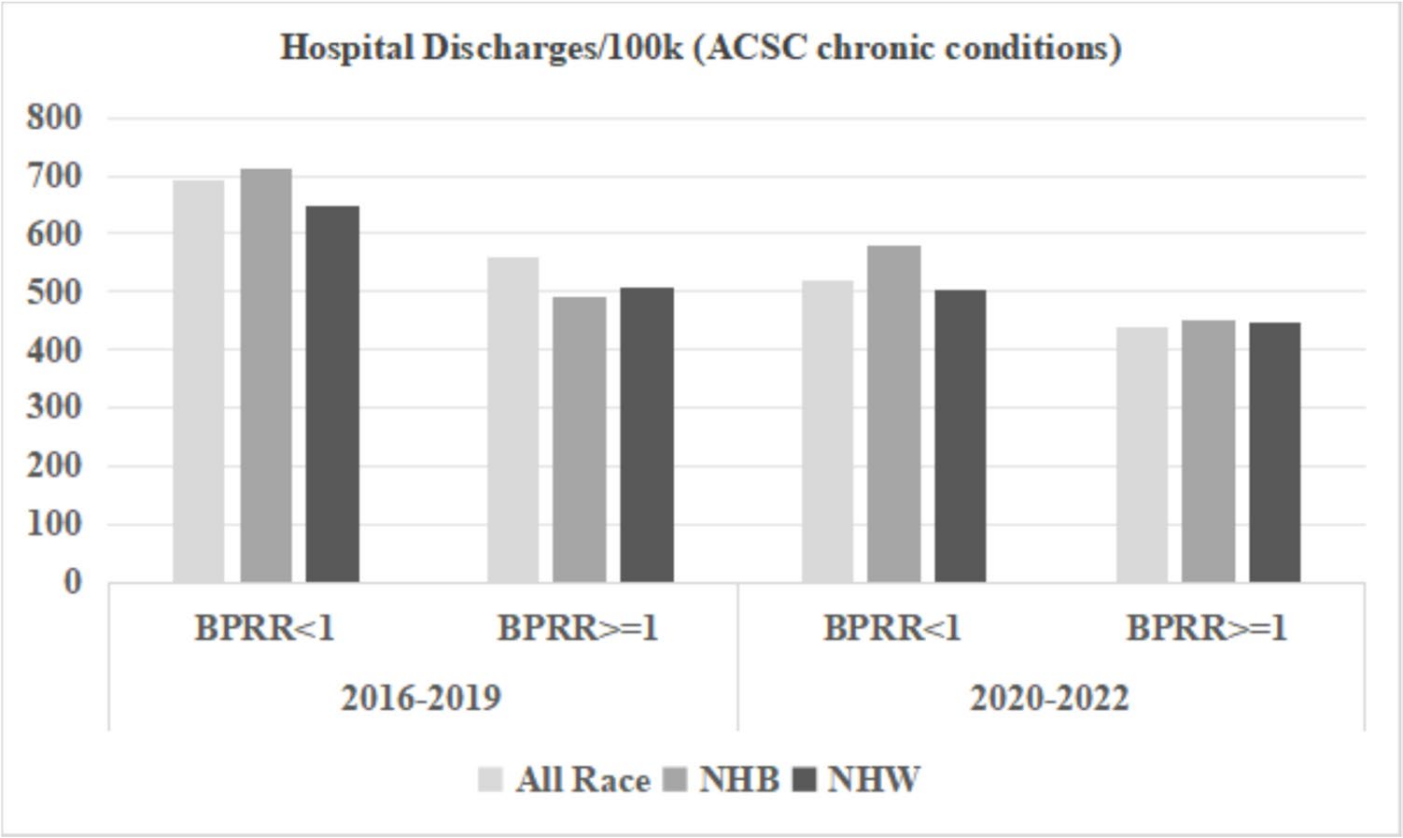
Yearly hospital discharges due to chronic Ambulatory Care Sensitive Conditions (ACSC) per 100,000 people in Georgia counties, 2016–2019 and 2020–2022. BPRR, Black physician representativeness ratio; NHB, non-Hispanic Black; NHW, non-Hispanic White

**Fig. 4:**
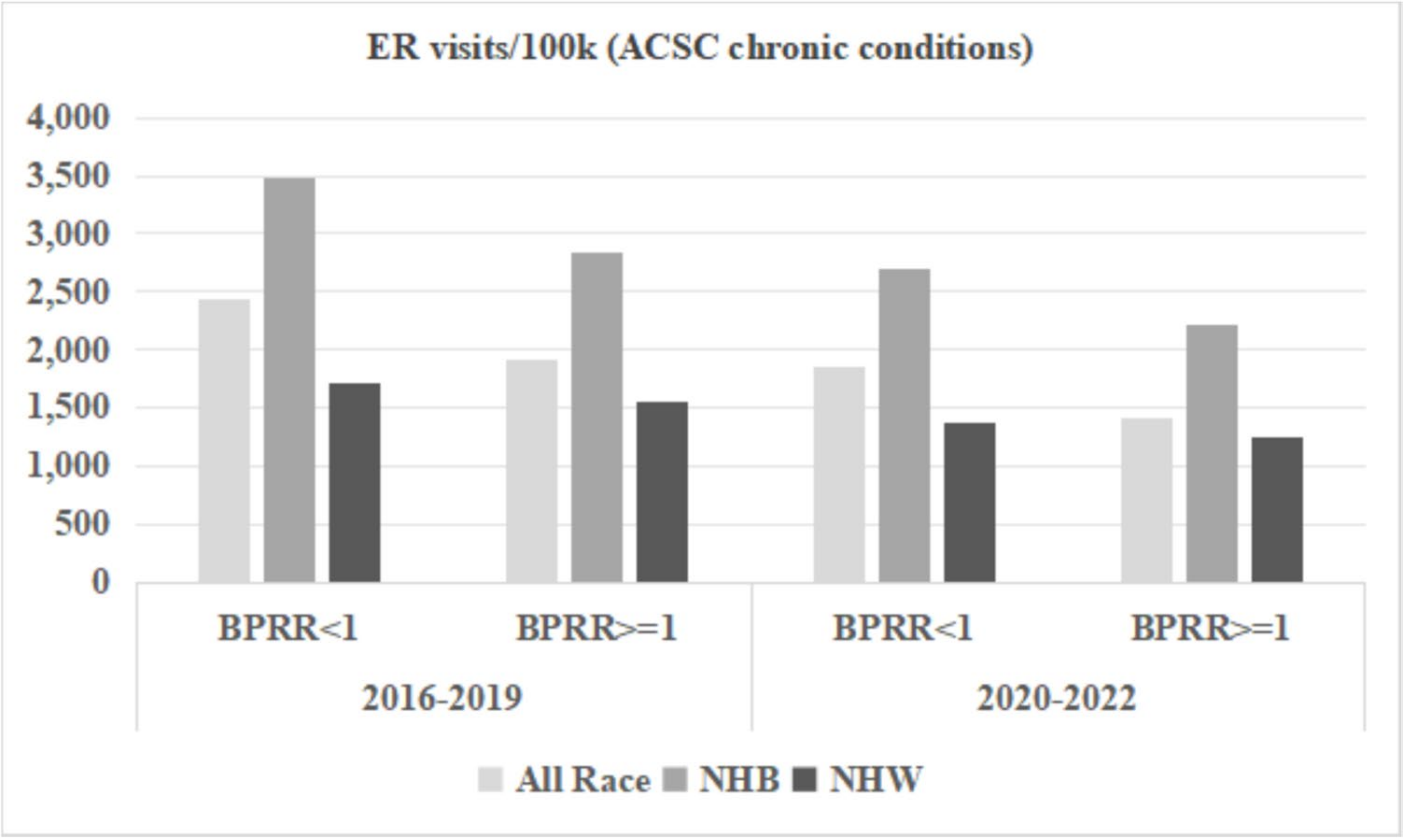
Yearly Emergency Room visits due to chronic Ambulatory Care Sensitive Conditions (ACSC) per 100,000 people in Georgia counties, 2016–2019 and 2020–2022. BPRR, Black physician representativeness ratio; NHB, non-Hispanic Black; NHW, non-Hispanic White

**Fig. 5:**
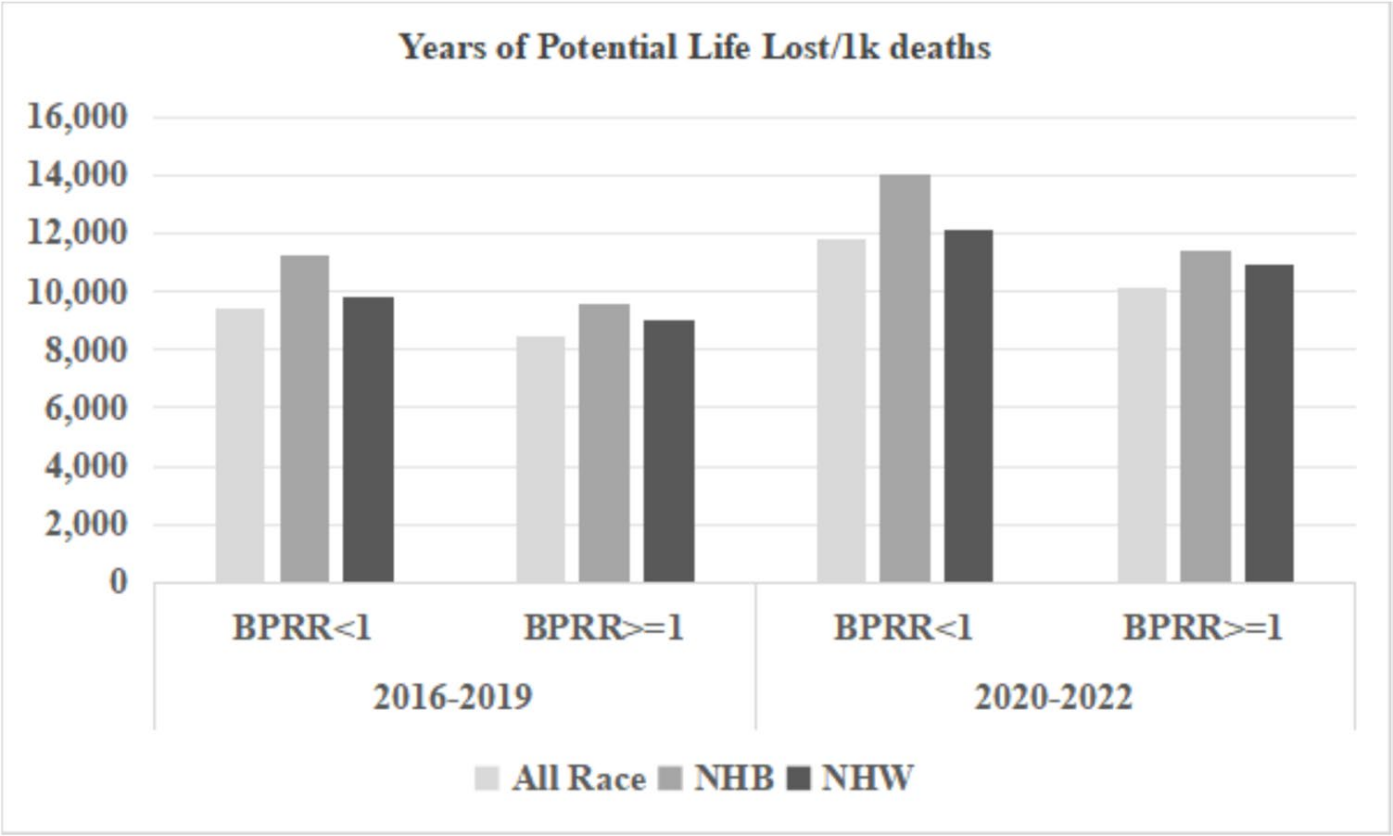
Yearly years of potential life lost per 1,000 deaths in Georgia counties, 2016–2019 and 2020–2022. BPRR, Black physician representativeness ratio; NHB, non-Hispanic Black; NHW, non-Hispanic White

**Fig. 6:**
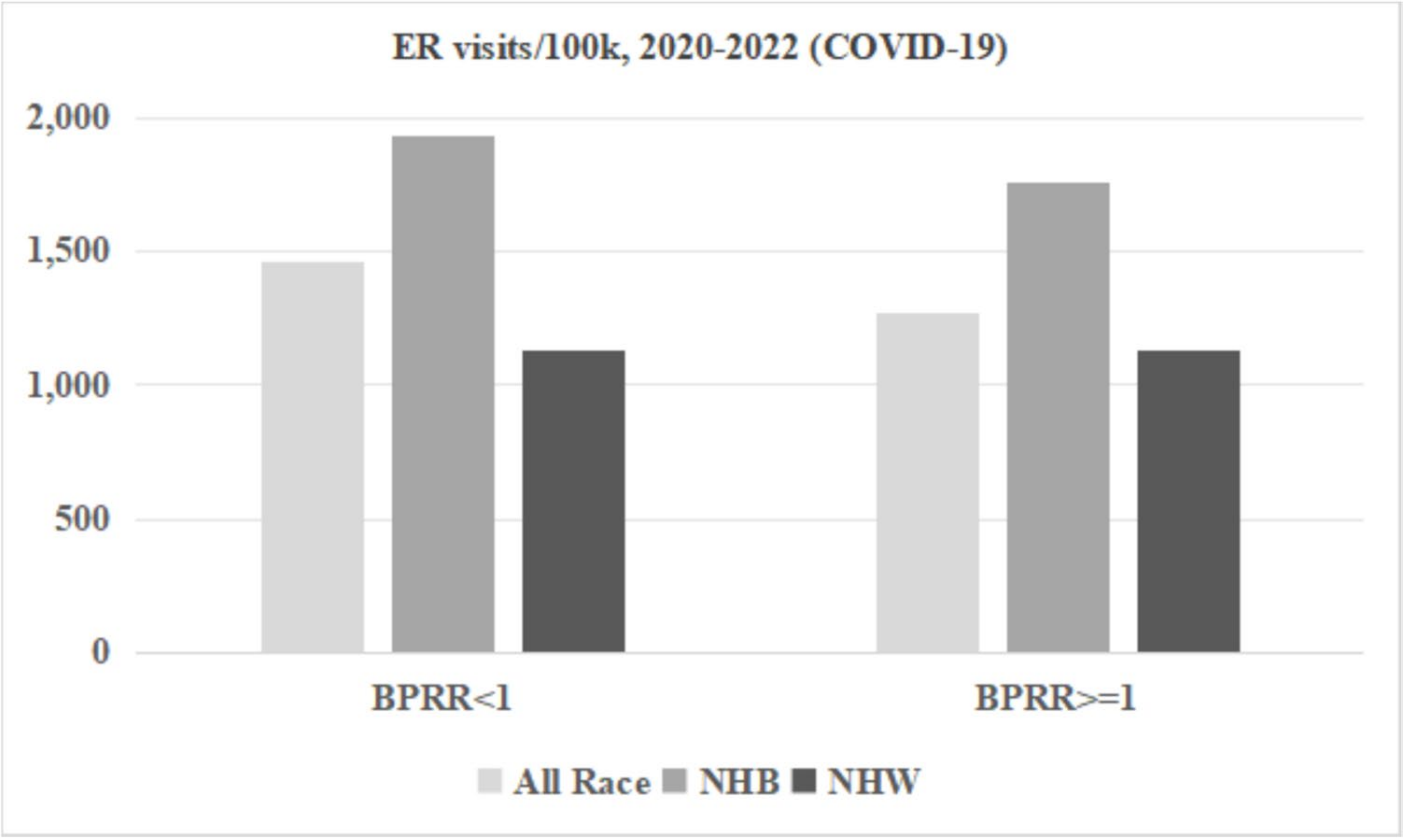
Yearly hospital discharges due to COVID- 19 per 100,000 people in Georgia counties, 2020–2022. BPRR, Black physician representativeness ratio; NHB, non-Hispanic Black; NHW, non-Hispanic White

**Fig. 7:**
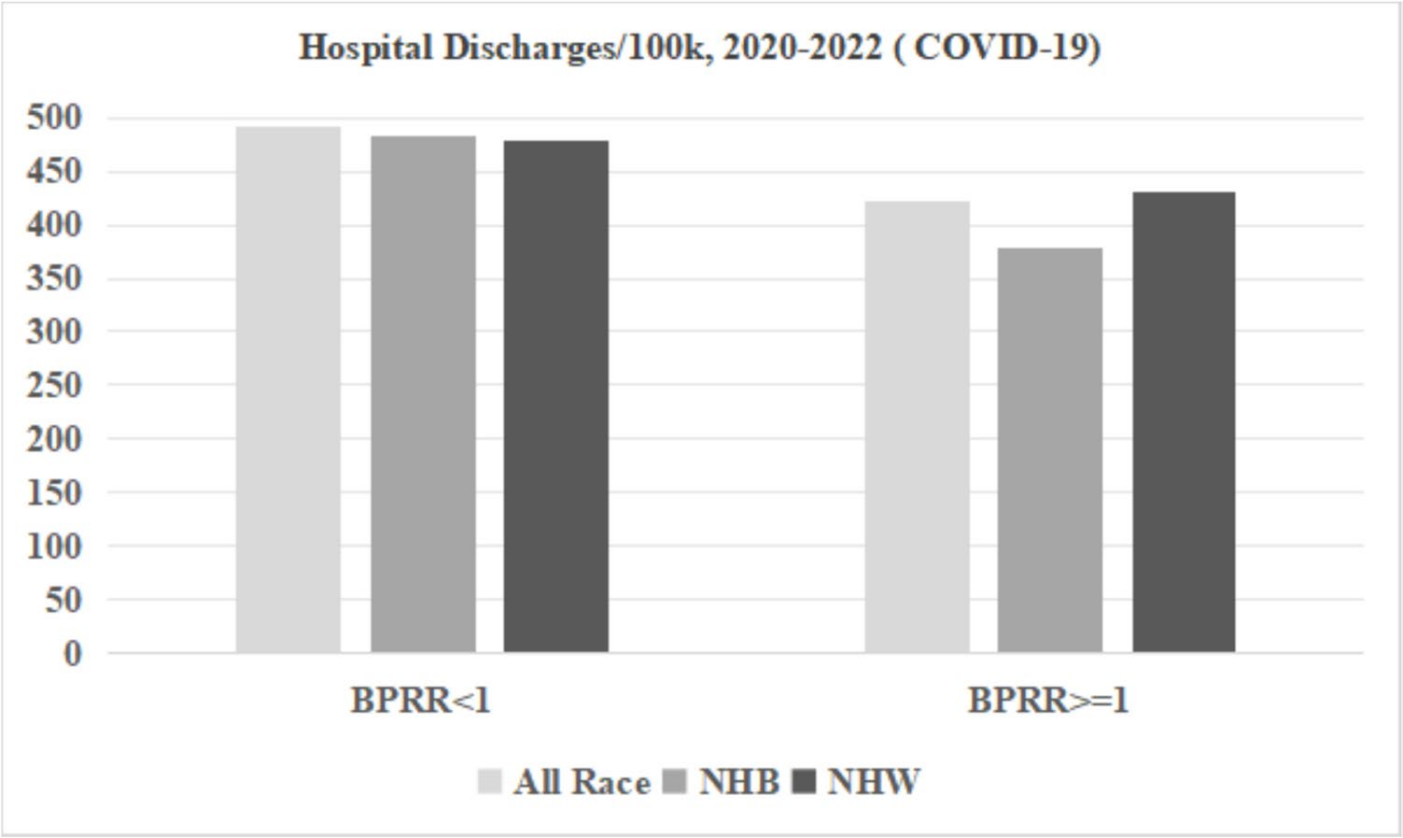
Yearly emergency room visits due to COVID- 19 per 100,000 people in Georgia counties, 2020–2022. BPRR, Black physician representativeness ratio; NHB, non-Hispanic Black; NHW, non-Hispanic White

Figures 3, 4, and 5 display the ER visits and hospital discharges attributed to chronic ACSC conditions and YPLL for the NHB population, NHW population, and the entire population for both study periods. Rates of ER visits and hospitalizations due to chronic ACSC conditions declined between 2016–2019 and 2020–2022, while YPLL increased for all race groups in counties below and above the physician representativeness threshold across the two time periods. Disparities between NHB and NHW populations were smaller in counties with BPRR ≥ 1 for all outcomes for both time periods. Figures 6 and 7 illustrate the outcomes due to COVID- 19 for NHB populations, NHW populations, and the entire population during 2020–2022. In the counties with BPRR ≥ 1, the disparity in ER visits due to COVID- 19 between the NHB and NHW got smaller, and the NHB population even had lower hospitalization rate due to COVID- 19 than the NHW population.

The regression model results for chronic ACSC hospital discharges and ER visits, and YPLL during 2020–2022 are shown in Table 3. The counties with BPRR ≥ 1 had a smaller NHB-NHW disparity in yearly hospital discharge rates before or after adjustment for the covariates, but the difference was not statistically significant (Unadjusted: estimate =  − 67.5/100,000, *p*-value = 0.169; adjusted: estimate =  − 65.0/100,000, *p*-value = 0.225). The counties with BPRR ≥ 1 had reduced disparity in yearly ER visit rate with borderline significance (estimate =  − 356.6/100,000, *p*-value = 0.073), but the difference was attenuated to almost 0 after adjustment for covariates (estimate = 0.1/100,000, *p*-value = 0.999). The counties with BPRR ≥ 1 had significantly reduced disparities in yearly YPLL rate in both unadjusted and fully adjusted models (Unadjusted: estimate =  − 1467.9/1000, *p*-value = 0.037; adjusted: estimate =  − 1658.4/1000, *p*-value = 0.041).

**Table 3:** Unadjusted and adjusted associations between county-level BPRR and Black-White differences in the yearly hospital discharges rate, ER visits rate due to chronic ACSC, and years of potential life lost/1,000 deaths, 2020–2022

		Unadjusted			Adjusted		
		Estimate	95% CI	*p*-value	Estimate	95% CI	*p*-value
NHB-NHW hospital discharges/100 k people							
BPRR	< 1	ref			ref		
	≥ 1	− 67.5	− 164.1 to 29.1	0.169	− 65.0	− 170.6 to 40.7	0.225
NHB-NHW ER visits/1,000 people							
BPRR	< 1	ref			ref		
	≥ 1	− 356.6	− 746.9 to 33.8	0.073	− 0.1	− 392.2 to 392.0	0.999
NHB-NHW years of potential life lost before age 75/1000 deaths							
BPRR	< 1	ref			ref		
	≥ 1	− 1467.9	− 2846.2 to − 89.7	0.037	− 1658.4	− 3247.3 to − 69.5	0.041

*BPRR* Black physician representative ratio, *ER* emergency room, *CI* confidence interval, *NHB* non-hispanic black, *NHW* non-hispanic white

Covariates for adjustment included the proportion of the population that was Black, median age, proportion of people living under the federal poverty line, proportion of people without health insurance, rural status, and whether the county had at least one hospital.

In Table 4, regression results for COVID- 19 hospital discharges and ER visits were displayed. The counties with BPRR ≥ 1 had a reduced NHB-NHW disparity in yearly COVID hospital discharge rates before or after adjustment for the covariates, but the differences were not statistically significant (unadjusted: estimate =  − 59.1/100,000, *p*-value = 0.139; adjusted: estimate =  − 14.3/100,000, *p*-value = 0.737). Among counties with BPRR ≥ 1, the NHB-NHW disparity in yearly COVID- 19 ER visit rate was also smaller before adjustment (unadjusted: estimate =  − 174.5/100,000, *p*-value = 0.289), and the difference was attenuated with adjustment of covariates (adjusted: estimate =  − 27.9/100,000, *p*-value = 0.876).

**Table 4:** Unadjusted and adjusted association between county-level BPRR and Black-White differences in the yearly COVID- 19 hospital discharges rate and ER visits rate, 2020–2022

		Unadjusted			Adjusted		
		Estimate	95% CI	*p*-value	Estimate	95% CI	*p*-value
NHB-NHW COVID- 19 hospital discharges/100 k people							
BPRR	< 1	ref			ref		
	≥ 1	− 59.1	− 137.7 to 19.4	0.139	− 14.3	− 98.5 to 69.9	0.737
NHB-NHW COVID- 19 ER visits/1000 people							
BPRR	< 1	ref			ref		
	≥ 1	− 174.5	− 498.8 to 149.8	0.289	− 27.9	− 382.8 to 326.9	0.876

*BPRR* Black physician representative ratio, *ED* emergency department, *CI* confidence interval, *NHB* non-Hispanic Black, *NHW* non-hispanic white

Covariates for adjustment included proportion of population that were Black, median age, proportion of people living under federal poverty line, proportion of people without health insurance, rural status, and whether the county had at least one hospital.

## Discussion

Counties in Georgia with higher Black physician representativeness (BPRR ≥ 1) had better overall health outcomes for the total population. Specifically, counties with BPRR ≥ 1 had lower rates of hospital discharges and ED visits for ACSCs and lower YPLL for both NHB and NHW populations. The trend of higher Black physician representation and improved health outcomes supports previous findings that Black physician representativeness was associated with decreased mortality and improved life expectancy for the Black population [5, 6, 15]. These findings suggest that a more representative physician workforce may contribute to better management of chronic conditions, improved preventive care, and reduced premature mortality for all patients.

This study adds to the literature by documenting the relationship between Black physician representativeness, health, and health equity during a global pandemic. The COVID- 19 pandemic exacerbated existing health inequities, disproportionately affecting Black communities in Georgia [16]. Counties with BPRR ≥ 1 had lower rates of hospitalizations and ER visits and fewer YPLL before the COVID- 19 pandemic and this study found that the impact of Black physician representativeness on these outcomes was particularly pronounced during the pandemic, with statistically significant reductions in YPLL inequities between NHB and NHW populations. The inequities in hospital discharge rates, ED visit rates, and YPLL were relatively narrower in these counties, particularly during the COVID- 19 pandemic. In our analysis, the adjustment for the covariates considerably attenuated the impact of BPRR on the NHB-NHW disparity in ER visits, but not the hospital discharge or the years of potential life lost. This finding indicates Black physician representativeness may affect the three outcomes through different mechanisms, which would be worth exploring in the future studies.

These findings align with previous research showing that racial concordance between patients and physicians can improve communication, trust, and adherence to medical advice, thereby reducing health inequities, underscoring the critical role of physician representation in mitigating the effects of public health crises and ensuring equitable healthcare delivery [23]. Black physician representation improves health outcomes through mechanisms likely including enhanced patient-provider communication, increased trust and adherence to medical recommendations, and culturally competent care. Additionally, Black physicians may advocate more effectively for policy changes and resources that benefit the communities where they practice. The presence of Black physicians can also potentially inspire future generations to pursue careers in medicine, further enhancing workforce diversity and health equity [15, 24].

Georgia has the second largest uninsured population in the U.S. and ranked 45 th in the Commonwealth Fund’s 2023 Scorecard on State Health System Performance [25]. Georgia falls in the lowest quartile for healthcare quality and in the 3rd quartile for healthcare disparities in the Agency for Healthcare Research & Quality (AHRQ) 2023 National Healthcare Quality and Disparities Report [22]. Georgians have higher than average rates of diabetes, hypertension, and chronic kidney disease, and the state ranks 48 th for rates of low-birth-weight births [26]. Georgia has above average rates of avoided healthcare due to costs, lower than average rates of people reporting having a regular source of healthcare, and shortages of primary care, mental health, and dental care providers [26]. In Georgia, where health outcomes are poor, and the healthcare workforce is limited, increasing Black physician representation in the physician workforce could potentially mitigate these disparities.

In this study, 19.1% of physicians identified as Black or African American, which is higher than the national average, yet still fails to reflect the state’s Black population of 33%. Georgia is home to one of the four HBCU medical schools in the nation. This institution plays a crucial role in training Black physicians and diversifying the state’s healthcare workforce. These HBCU medical schools produce culturally competent healthcare providers committed to serving the underserved communities [13]. Although there are only four HBCU medical schools in the country, collectively they have graduated more Black physicians in a 10-year period (2000–2020) than the top 10 predominantly White medical schools combined [27]. The ongoing support for and expansion of HBCU medical schools can be a pivotal strategy to build a representative workforce and promote health equity [27–29]. In the context of Georgia’s physician shortage and large rural population, our findings suggest that Black physicians could play a crucial role in improving health outcomes for all patients by increasing access to care and reducing the need for emergency services through preventive and primary care.

Our findings have important implications for healthcare policy and practice. Efforts to increase the representation of Black physicians should continue to be prioritized as a strategy to improve population health and reduce inequities. State and federal policymakers should support initiatives that enhance diversity in medical education and address barriers to entry and workforce sustainability for underrepresented minorities in the medical profession. Costs of medical education continue to increase and Black medical trainees are more likely to have premedical education and consumer debt than their white and Asian counterparts [30]. Service-based student loan repayment programs are increasingly being used to increase the physician workforce in underserved communities and may influence where physicians practice and what specialty they choose [31]. Another promising approach involves pathway programs for youth to learn about and experience healthcare professions and targeted medical school recruitment from community colleges, HBCUs, and institutions that serve large minoritized student populations [32]. Finally, strategies are needed to support minoritized physicians already in the healthcare workforce. Black physicians and those who identify as two or more races were most affected by burnout during the COVID- 19 pandemic [33]. Black physicians reported higher rates of workplace discrimination from both patients and colleagues than their peers [33]. Policymakers and health systems should implement policies and practices that promote racial concordance, provider well-being and burnout prevention, and culturally tailored care [34].

Several limitations should be noted. First, the study’s cross-sectional nature precludes causal inferences. Second, our measure of Black physician representation may not capture the full complexity of healthcare access and quality. Third, excluding counties with fewer than five physicians may bias results. Fourth, unmeasured confounders such as physician quality and patient preferences could influence the observed associations. Fifth, the BPRR measure was based on the survey data collected during 2019–2020. The actual Black physician representativeness in the counties may not stay on the same level before or after this time period. Sixth, the core counties of the Atlanta metro area (Fulton/Dekalb/Cobb/Gwinnett/Fayette), which presumably have the highest concentration of health promoting/preserving resources, all had a BPRR < 1, potentially indicating that the effect of physician representation is underestimated in our study. Finally, it is unknown if the observed ecological associations would apply on the individual level; i.e., we do not know if greater representation at the county level led to greater racial concordance of patients with physicians which in turn influenced patient outcomes. Further research is needed to explore the long-term impact of Black physician representation on health outcomes and to identify the most effective strategies for increasing diversity in the healthcare workforce [7]. It is unknown whether the results would be generalizable to other states, but future studies should investigate representation of Black physicians, as well as other under-represented groups of physicians in other states. Longitudinal studies and qualitative research can provide deeper insights into how physician representation influences health outcomes and inform targeted interventions to promote health equity.

## Conclusion

This study demonstrates that higher Black physician representation in the county physician workforce is associated with improved health outcomes and reduced racial health disparities in Georgia. These findings underscore the potential importance of diversity in the healthcare workforce and the need for policies supporting Black physicians’ training and retention. By addressing the critical issue of physician workforce representation, we may make strides toward achieving health equity and improving population health.
